# Immediate effects of cigar smoking on respiratory mechanics and exhaled biomarkers; differences between young smokers with mild asthma and otherwise healthy young smokers

**DOI:** 10.1186/s12971-016-0095-6

**Published:** 2016-08-18

**Authors:** Andreas S. Lappas, Efstathia M. Konstantinidi, Anna S. Tzortzi, Chara K. Tzavara, Panagiotis K. Behrakis

**Affiliations:** 1 George D Behrakis Research Lab, Hellenic Cancer Society, 8 Doryleou Str, P.C. 11521 Athens, Greece; 2 Biomedical Research Foundation, Academy of Athens, Athens, Greece; 3 Institute of Public Health, American College of Greece, Athens, Greece; 4 Department of hygiene, epidemiology and medical statistics, National and Kapodistrian University of Athens, Medical School, Athens, Greece

## Abstract

**Background:**

We aimed to investigate the immediate respiratory effects of cigar smoking(CS), among young smokers with and without mild asthma.

**Materials and methods:**

Forty-seven young smokers (18–31years old, 29 males, average pack-years = 3.6 ± 2.8) were enrolled. Twenty-two were mild asthmatics(MA-subgroup) and the remaining 25 were otherwise healthy smokers(HS-subgroup). Exhaled carbon monoxide(eCO), multi-frequency respiratory system impedance(Z), resistance(R), reactance(X), frequency-dependence of resistance(fdr = R5Hz - R20Hz), resonant frequency(f_res_), reactance area(AX) and exhaled nitric oxide(FENO) were measured at the aforementioned sequence, before and immediately after 30 min of CS, or equal session in the smoking area while using a sham cigar(control group). Chi-square, student’s t-tests, mixed linear models and Pearson correlation tests were used for the statistical analysis; level of significance was defined as *p* < 0.05.

**Results:**

Immediately after CS, Z5Hz, R5Hz, R10Hz, R20Hz and eCO increased significantly in both subgroups(MA and HS). A greater increase was found for R20 in HS-subgroup. Fdr, f_res_ and AX increased in MA, while decreased in HS. On the contrary, X10 decreased in MA and increased in HS, while X20 showed a greater decrease in MA. Changes in fdr, f_res_ and AX were significantly correlated in both subgroups. No significant FENO alterations were detected in both subgroups.

**Conclusion:**

CS has immediate effects on pulmonary function. Mild asthma predisposes to higher increase of peripheral resistance(increased fdr). In otherwise healthy smokers, central resistance(R20Hz) is more affected. FENO levels are not significantly affected by CS.

## Background

Immediate respiratory effects of cigarette, e-cigarette and water-pipe smoking have been adequately investigated [1–4]. For cigar smoking(CS), however, there is relative lack of such studies. Recent data regarding long-term effects of CS on pulmonary function, detect that CS is associated with decreased values of FEV_1_ and FEV_1_/FVC, and increased odds of airflow obstruction, even in subjects who have never smoked cigarettes [5]. Therefore, we hypothesized that there are also immediate respiratory effects, even after a single cigar smoking.

Furthermore, smoking population includes people with respiratory co-morbidities. Asthma is the most prevalent of them among young smokers and it has been shown that approximately 30 % of adults with asthma are current smokers [6]. Cigarette smoking is known to be responsible for accelerated decline in lung function among asthmatics [7] and poorer control of asthma symptoms [8]. Furthermore, cigarette smoking has been proved to have more deleterious immediate inflammatory effects in asthmatic smokers compared to healthy smokers [9]. Based on the above, we found interesting to test the hypothesis that the acute respiratory system response to CS would be different among otherwise healthy smokers and smokers with mild asthma, considering also that CS is a more intense stimuli than cigarette smoking [10].

Our experimental design was focused on young smokers because CS is popular amongst the youth, with a reported prevalence of 12.6 % among high school students in the USA [11]. Moreover, young smokers are more vulnerable to unrealistic perceptions regarding safety of CS, used as advertisement tools from tobacco industry. It has been shown that only 8.7 % of cigar smokers consider themselves to be at high risk of cancer development, while the glamorized image of cigar smokers presented in the media appears to be accepted both by those who smoke cigars and those who do not [10].

Through this study, we aimed to detect the immediate respiratory effects of CS and, particularly, to investigate the possibility of a different acute response of the respiratory system among young smokers with and without mild asthma.

## Methods

### Subjects

Forty-seven young adults (29 males, average age = 23.4 ± 4.2 y, range = 18–31y) voluntarily participated in the study. Twenty-two of them were mild asthmatics, being recruited from our outpatients’ lung function clinic(MA-subgroup). All twenty-two asthmatics were sporadically treated with short-acting β_2_-agonists. They all reported atopic history (20 with allergic rhinitis, five with allergic conjunctivitis and two with atopic dermatitis), mild dry cough, chest tightness and wheezing. All symptoms were sporadic, elicited by exposure to certain substances, or following respiratory infections. All 22 MA-subjects completed the Asthma Control Questionnaire(ACT) and reported an ACT-score ≥20. Furthermore, they were all free of symptoms and any medication at the time of the study (conducted out of pollen season), and for the past 4 weeks. The remaining 25 subjects were otherwise healthy smokers(HS-subgroup).

All 47 subjects were current cigarette smokers (reported smoking of at least one cigarette during the past 30 days [12–14], average cigarette consumption was 3.6 pack-years) and frequent cigar smokers (approximate consumption = 1 cigar/week). Exclusion criteria for all subjects included any kind of diseases (even a common cold during the previous 2 weeks) with the exception of mild asthma in MA-subgroup, pregnancy, lactation and current use of any medication.

### Study design

A crossover, laboratory-based study design was applied on the abovementioned subpopulations, in experimental and control sessions, which the participants underwent one at a time. During experimental session, each of the 47 subjects was instructed to remain in sitting position and smoke a single cigar ad lib for 30 min inside a special smoking area (3.6 m × 3 m × 2.7 m = 29.16 m^3^) with the door closed and the window opened. Each participant smoked approximately half of a large cigar (9 g weight, 150 mm length, 13 mm diameter)^1^. For control purposes, each of the 47 subjects used a sham cigar of the same size ad lib for 30 min, under the above mentioned conditions. Since there was no smoke production by the use of the sham cigar, blind control was impossible.

In line with relevant, previously published methodology, all 47 subjects were instructed to avoid consuming food, drinks, beverages 4 h prior to each session [1–4]. In regard to the time of smoking abstinence prior to the measurements, there is considerable inconsistency in the literature, ranging from hours to days [15–17]. Furthermore, the time frame for detecting the short-term effects of CS on lung function is unclear. We chose abstinence from both cigarette and cigar smoking for 12 h prior to the measurements, which has been previously used in studies with relevant objectives and methodology [9]. Furthermore, this time frame secured the good compliance of the subjects to abstinence from smoking. Each session began at 8 am and each participant was advised to avoid smoking between 8 pm and 8 am. This setting was easily achievable by all our subjects, who were mild smokers.

The ethics committee of the Hellenic Cancer Society, Athens, Greece, provided ethics approval (protocol number: 561/28-1-14). Each subject read and signed an informed consent form prior to study enrollment.

### Pulmonary function assessment

Spirometry was performed at an initial session (day 1), along with the assessment of the subjects’ medical history (including ACT in the MA-subgroup). In the second (control) and the third (experimental) session, measurement of the exhaled carbon monoxide(eCO), impulse oscillometry(IOS) and measurement of fractional exhaled nitric oxide(FENO) were performed in the above mentioned sequence, before and immediately after the corresponding intervention. All three sessions took place in three consecutive days and the control session (day 2) always preceded the experimental session (day 3) for consistency purposes.

(i) *Spirometry and flow-volume loop*
  Dynamic expiratory lung volumes and flows (FVC, FEV_1_, FEV_1_/FVC %, PEF, and FEF at 25, 50, and 75 % of exhaled VC) were measured in sitting position with a nose-clip applied, using a Jaeger MasterScreen spirometry system in all subjects according to the recommendations of the ATS/ERS task force guidelines [18]. Predicted values of the abovementioned parameters were calculated by the software of the equipment used.

(i) *eCO measurements*
  Measurements were performed with the Bedfont “Microsmokerlyser” equipment, according to the operating instructions provided by the manufacturer: a nose-clip was applied on the subjects, and they were instructed to quietly inhale and hold their breath for approximately 15 s, and consecutively quietly exhale for approximately 10 s.
(ii) *IOS measurements*
  Respiratory system total impedance at 5 Hz(Z5), resistance at 5, 10, and 20 Hz (R5, R10, and R20), frequency dependence of resistance(fdr = R5-R20), reactance at 5, 10, and 20 Hz (X5, X10, and X20), resonant frequency(f_res_) and reactance area(AX) were measured with the use of the Viasys Jaeger Masterscreen IOS system (heated pneumotach, resistance = 0.05 kPa/(L/s) at 10 L/s), according to the ERS task force guidelines [19]. Three reproducible consecutive trials (CV% < 10 %) were performed, of 90 s duration each.
(iii) *FENO measurements*
  Measurements were made in sitting position with a nose-clip applied, using an Eco Medics AG CLD 88 Series chemiluminescence analyzer equipped with a Spiroware 3.0 software program. Subjects were instructed to inhale deeply through a filtered mouthpiece and consecutively exhale at a mouth flow rate of approximately 50 mL/s for 10 s. Expiratory flow was held approximately steady by applying a constant positive pressure(10 cmH_2_O) through a resistance factor, while instructing the patient to exhale steadily using visual stimulation on the system screen. Three reproducible consecutive trials(CV% < 10 %) were performed with a 30 s. interval.

### Statistical analysis

Quantitative variables were expressed as mean values (SD), while qualitative variables were expressed as absolute and relative frequencies. For the comparison of proportions chi-square tests were used. Student’s t-tests were used for the comparison of spirometric data, baseline impulse oscillometry(IOS) and fractional exhaled nitric oxide(FENO) measurements between young subjects with mild asthma(MA) and healthy smokers(HS). Pearson correlation coefficients were computed in order to explore the association of changes in IOS measurements after cigar smoking.

In order to evaluate the effect of smoking, the effect of mild asthma, and the interaction effect of time (pre vs. post measurements) with smoking (cigar smoking or control session) mixed linear models were used [20, 21]. In order to investigate if changes before and after the smoking session were different between the group with mild asthma (MA) and the group of healthy smokers (HS), the interaction effect of time with the presence of asthma was tested in mixed linear models that referred only to smoking session. Regression coefficients (β) with standard errors (SE) were computed from the results of the models.

In each of the mixed linear models, each outcome of impulse oscillometry(IOS), fractional exhaled nitric oxide(FENO) and exhaled carbon monoxide(eCO) measurements represented a single dependent variable, and it was tested simultaneously for the effect of time, asthma and smoking, along with the interaction effects.

All reported p values are two-tailed. Statistical significance was set at *p* < 0.05 and analyses were conducted using STATA statistical software(version 11.0).

## Results

Demographics and baseline spirometric data are presented in Table 1. MA and HS subgroups were similar in terms of sex, age, BMI and pack-years index(Table 1). Asthmatic smokers presented with significantly lower values of FEV_1_, FEV_1_/FVC, FEF_25%-75%_, FEF_25%_, FEF_50%_ and FEF_75%_ compared with healthy smokers. Baseline IOS, FENO and eCO measurements are presented in Table 2. Significantly higher baseline values of Z5, R5, R10, R20, X5 and FENO were found in asthmatics in both experimental and control sessions. f_res_, X10 and AX differed between MA and HS subgroup at baseline measurements of control session.

**Table 1:** Demographics and baseline spirometric data for young smokers with mild asthma (MA subgroup) and healthy young smokers (HS subgroup)

	MA subgroup	HS subgroup	
	(N = 22)	(N = 25)	
	Mean (SD)	Mean (SD)	*P*
Sex, N (%)			
Males	12 (54.5)	17 (68.0)	0.344*
Females	10 (45.5)	8 (32.0)	
Age (years)	23.6 (3.9)	23.2 (4.5)	0.525**
BMI (kg/cm^2^)	23.9 (2.9)	23 (3.4)	0.404**
Pack-years	3.6 (2.6)	3.6 (2.9)	0.747**
FVC (% pred.)	97.75 (10.6)	105.5 (10.8)	0.013**
FEV1 (% pred.)	90.58 (9.4)	109.5 (9.2)	<0.001**
FEV1/FVC (% pred.)	79 (5.6)	88.8 (5.3)	<0.001**
PEF (% pred.)	91.49 (12.3)	99 (13.1)	0.062**
FEF_25%-75%_ (% pred.)	71.44 (13.9)	112.7 (22.3)	<0.001**
FEF_25%_ (% pred.)	85.8 (16.6)	111 (16.6)	<0.001**
FEF_50%_ (% pred.)	76.46 (14.6)	114 (19.9)	<0.001**
FEF_75%_ (% pred.)	68.63 (20.0)	118 (39.6)	<0.001**

*Pearson’s chi-square test; **Student’s *t*-test

**Table 2:** Baseline impulse oscillometry (IOS), fractional exhaled nitric oxide (FENO) and exhaled carbon monoxide (eCO) measurements in young subjects with mild asthma (MA) and healthy smokers (HS), in experimental (smoking) and control sessions separately

	MA (*N* = 22)	HS (*N* = 25)	
	Mean (SD)	Mean (SD)	P*
Z5 [kPa/(L/s)]			
Exrerimental group	0.41 (0.13)	0.30 (0.06)	<0.001
Control group	0.42 (0.13)	0.31 (0.07)	0.001
R5 [kPa/(L/s)]			
Exrerimental group	0.39 (0.12)	0.28 (0.06)	<0.001
Control group	0.40 (0.13)	0.29 (0.07)	0.001
R10 [kPa/(L/s)]			
Exrerimental group	0.36 (0.11)	0.26 (0.05)	<0.001
Control group	0.33 (0.11)	0.27 (0.07)	0.001
R20 [kPa/(L/s)]			
Exrerimental group	0.35 (0.10)	0.26 (0.05)	<0.001
Control group	0.36 (0.10)	0.27 (0.08)	0.002
fdr [kPa/(L/s)]			
Exrerimental group	0.04 (0.06)	0.03 (0.03)	0.407
Control group	0.04 (0.06)	0.02 (0.04)	0.102
f_res_ (Hz)			
Exrerimental group	11.84 (3.35)	10.48 (2.75)	0.133
Control group	12.83 (4.73)	10.31 (2.43)	0.024
X5 [kPa/(L/s)]			
Exrerimental group	−0.12 (0.05)	−0.09 (0.03)	0.014
Control group	−0.13 (0.05)	−0.10 (0.03)	0.015
X10 [kPa/(L/s)]			
Exrerimental group	−0.02 (0.05)	0.0004 (0.03)	0.070
Control group	−0.02 (0.05)	0.004 (0.03)	0.026
X20 [kPa/(L/s)]			
Exrerimental group	0.08 (0.05)	0.09 (0.05)	0.325
Control group	0.09 (0.08)	0.10 (0.05)	0.643
AX (kPa/L)			
Exrerimental group	0.42 (0.48)	0.23 (0.17)	0.072
Control group	0.47 (0.49)	0.23 (0.15)	0.029
FENO (ppb)			
Exrerimental group	22.50(15.6)	12.00 (6.9)	0.004
Control group	19.84 (14.6)	10.57 (4.7)	0.004
eCO (ppm)			
Exrerimental group	3.68 (2.01)	4.40 (2.31)	0.265
Control group	4.14 (2.12)	3.84 (2.27)	0.647

*Student’s *t*-test

Table 3 shows mean changes of IOS, FENO and eCO measurements before and immediately after 30 min of cigar smoking or control session separately in MA and HS subgroups.

**Table 3:** Mean changes of impulse oscillometry (IOS), fractional exhaled nitric oxide (FENO) and exhaled carbon monoxide (eCO) measurements before and immediately after 30 min of cigar smoking (experimental session) or control session separately in young smokers with mild asthma (MA) and healthy smokers (HS)

	MA (*n* = 22)	HS (*n* = 25)
	Change (%)	Change (%)
Z5 [kPa/(L/s)]		
Exrerimental group	+0.04 (+10.6 %)	+0.03 (+8.8 %)
Control group	−0.02 (−4.0 %)	−0.01 (−2.6 %)
R5 [kPa/(L/s)]		
Exrerimental group	+0.05 (+11.5 %)	+0.03 (+10.4 %)
Control group	−0.02 (−4.1 %)	−0.01 (−2.7 %)
R10 [kPa/(L/s)]		
Exrerimental group	+0.03 (+8.3 %)	+0.03 (+12.0 %)
Control group	−0.02 (−5.1 %)	−0.01 (−2.3 %)
R20 [kPa/(L/s)]		
Exrerimental group	+0.02 (+4.8 %)	+0.04 (+16.6 %)
Control group	−0.01 (−3.8 %)	−0.003 (−1.0 %)
fdr [kPa/(L/s)]		
Exrerimental group	+0.03 (+72.1 %)	−0.01 (−50.0 %)
Control group	−0.003 (−6.5 %)	−0.01 (−26.5 %)
f_res_ (Hz)		
Exrerimental group	+1.55 (+13.1 %)	−0.27 (−2.6 %)
Control group	+0.17 (+1.3 %)	−0.03 (−0.3 %)
X5 [kPa/(L/s)]		
Exrerimental group	−0.04 (+3.4 %)	+0.003 (−3.4 %)
Control group	+0.002 (−1.8 %)	−0.002 (+1.7 %)
X10 [kPa/(L/s)]		
Exrerimental group	−0.02 (+76.6 %)	+0.005 (+1200 %)
Control group	−0.003 (+13.5 %)	0,00 (0,0 %)
X20 [kPa/(L/s)]		
Exrerimental group	−0.02 (−24.7 %)	−0.001 (−1.3 %)
Control group	−0.02 (−20.9 %)	−0.004 (−4.06 %)
AX (kPa/L)		
Exrerimental group	+0.15 (+35.4 %)	−0.013 (−6.0 %)
Control group	+0.04 (+7.5 %)	−0.003 (−1.2 %)
FENO (ppb)		
Exrerimental group	−2.41 (−10.7 %)	−0.52 (−4.4 %)
Control group	+0.38 (+1.9)	+0.35 (+3.4 %)
eCO (ppm)		
Exrerimental group	+11.36 (308.6 %)	+15.52 (+352.7 %)
Control group	+0.18 (+4.4 %)	+0.16 (+4.2 %)

Results from mixed linear models for IOS, FENO and eCO measurements are shown in Table 4. Significant changes from pre and post CS measurements were found for Z5, R5, R10, R20 and eCO. Also, a significant interaction effect of smoking with time was found for the aforementioned parameters, indicating that Z5, R5, R10, R20 and eCO increased significantly in both healthy and asthmatics but only after smoking and not in control session.

**Table 4:** Regression coefficients (β) and standard errors (SE) from mixed linear models for impulse oscillometry (IOS), fractional exhaled nitric oxide (FENO) and exhaled carbon monoxide (eCO) measurements, before and immediately after cigar smoking in healthy and mild asthmatic young smokers

	β for time (SE)	*P*	β for mild asthma (SE)	*P*	β for smoking (SE)	*P*	β for interaction of smoking with time (SE)	*P*
Z5 [kPa/(L/s)]	0.08 (0.04)	0.025	0.11 (0.02)	<0.001	0.01 (0.02)	0.703	−0.05 (0.02)	0.041
R5 [kPa/(L/s)]	0.09 (0.03)	0.012	0.11 (0.02)	<0.001	0.01 (0.02)	0.686	−0.05 (0.02)	0.024
R10 [kPa/(L/s)]	0.07 (0.03)	0.013	0.10 (0.02)	<0.001	0.01 (0.02)	0.536	−0.04 (0.02)	0.023
R20 [kPa/(L/s)]	0.07 (0.03)	0.019	0.08 (0.02)	<0.001	0.01 (0.01)	0.497	−0.04 (0.02)	0.038
fdr [kPa/(L/s)]	0.02 (0.02)	0.387	0.03 (0.01)	0.006	−0.003 (0.01)	0.737	−0.01 (0.01)	0.391
f_res_ (Hz)	1.10 (1.31)	0.400	2.46 (0.76)	0.001	0.37 (0.58)	0.523	−0.52 (0.83)	0.530
X5 [kPa/(L/s)]	−0.001 (0.01)	0.966	−0.03 (0.01)	0.001	−0.004 (0.01)	0.525	0.0004 (0.01)	0.964
X10 [kPa/(L/s)]	−0.01 (0.02)	0.575	−0.03 (0.01)	0.001	0.001 (0.01)	0.927	0.004 (0.01)	0.713
X20 [kPa/(L/s)]	−0.01 (0.02)	0.670	−0.02 (0.01)	0.062	0.01 (0.01)	0.534	−0.001 (0.01)	0.950
AX (kPa/L)	0.11 (0.15)	0.460	0.27 (0.10)	0.005	0.02 (0.07)	0.719	−0.05 (0.10)	0.614
FENO (ppb)	−3.18 (4.03)	0.431	9.47 (2.42)	<0.001	−2.06 (1.80)	0.253	1.77 (2.55)	0.488
eCO (ppm)	27.0 (2.36)	<0.001	−1.21 (2.72)	0.093	−0.09 (1.01)	0.933	−13.4 (1.43)	<0.001

Significant changes from pre and post cigar smoking measurements were found for Z5, R5, R10, R20 and eCO. Also, a significant interaction effect of smoking with time was found for the aforementioned parameters, indicating that Z5, R5, R10, R20 and eCO increased significantly in both healthy and asthmatics, but only after smoking and not in control session

eCO raised dramatically after smoking(up to 352.7 %) in both subgroups. Z5 increased by 8.8 % in HS and 10.6 % in MA. R5 raised by 10.4 % in HS and 11.5 % in MA. R10 raised by 12 % in HS and 8.3 % in MA. R20 increased by 16.6 % in HS and 4.8 % in MA. For all the aforementioned parameters, no significant differences in the degree of change was found between healthy and asthmatic smokers.

**Fig. 1:**
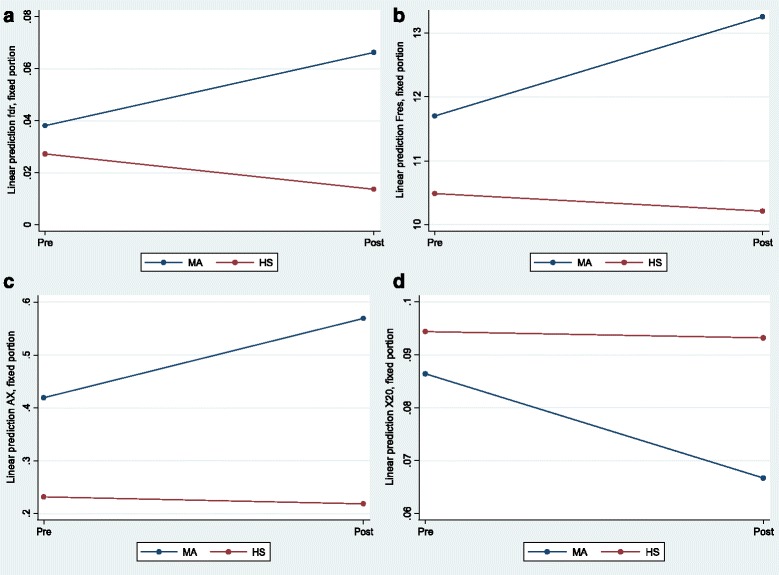
**a** Linear prediction of fdr before and after smoking session in the MA and HS group. **b** Linear prediction of f_res_ before and after smoking session in the MA and HS group. **c** Linear prediction of AX before and after smoking session in the MA and HS group. **d** Linear prediction of X20 before and after smoking session in the MA and HS group. MA = Mild Asthma, HS = Healthy Smokers

Table 5 presents the results from mixed linear models for significant findings concerning the differences in the degree of changes before and after smoking between healthy and asthmatics. A significant interaction of mild asthma with time was found for R20, fdr, f_res_, X10, X20 and AX. A greater increase was found for R20 in HS (+16.6 %) compared with MA (+4.8 %), *p* = 0.017. Fdr increased by 72.1 % in MA and decreased by 50 % in HS (*p* < 0.001). F_res_ increased by 13.1 % in MA and decreased by 2.6 % in HS (*p* = 0.007). Also, AX raised by 35.4 % in MA, whereas showed a decrease by 6 % in HS (*p* = 0.012). The aforementioned differences are illustrated in Fig. 1a, b and c.

**Table 5:** Regression coefficients (β) and standard errors (SE) from mixed linear models for significant findings concerning the differences in the degree of changes before and after cigar smoking between subjects with mild asthma (MA subgroup) and otherwise healthy smokers (HS subgroup)

	β for time (SE)	*P*	β for mild asthma (SE)	*P*	β for interaction of mild asthma with time (SE)	*P*
R20 [kPa/(L/s)]	0.04 (0.01)	<0.001	0.10 (0.02)	<0.001	−0.03 (0.01)	0.017
fdr [kPa/(L/s)]	−0.01 (0.004)	0.004	0.01 (0.02)	0.520	0.04 (0.01)	<0.001
f_res_ (Hz)	−0.27 (0.47)	0.556	1.21 (1.06)	0.252	1.83 (0.68)	0.007
X10 [kPa/(L/s)]	0.01 (0.004)	0.252	−0.02 (0.01)	0.173	−0.02 (0.01)	0.001
X20 [kPa/(L/s)]	−0.001 (0.01)	0.822	−0.01 (0.01)	0.583	−0.02 (0.01)	0.007
AX (kPa/L)	−0.01 (0.04)	0.767	0.19 (0.14)	0.184	0.16 (0.07)	0.012

A significant interaction of mild asthma with time was found for R20, fdr, fres, X10, X20 and AX. A greater increase after smoking session was found for R20 in healthy individuals. Fdr, fres and AX increased in asthmatics, while showed a decrease in healthy smokers. X10 decreased in asthmatics and increased in healthy smokers, while X20 had a greater decrease in asthmatics

X10 decreased in MA by 76.6 % and increased in HS subgroup by 1200 % (*p* = 0.001), while X20 showed a greater decrease in MA(−24.7 %) compared to HS(−1.3 %), *p* = 0.007 (Fig. 1d).

Changes in AX, fdr and f_res_ were significantly correlated in both MA and HS subgroups (*p* < 0.05) indicating that greater changes in one parameter are associated with greater changes in other IOS measurements.

FENO was not changed significantly after smoking, and no difference in the degree of FENO change was found between healthy and asthmatic smokers.

## Discussion

In this crossover study, we investigated for first time the immediate effects of CS on respiratory mechanics and exhaled biomarkers, among young regular cigarette and frequent cigar smokers with and without mild asthma. In order to investigate the effect of asthma on the acute respiratory system response to CS, we chose to include young smokers with mild, well controlled asthma (ACT ≥ 20), who had never been treated systematically, so as to avoid the influence of treatment and thereby investigate the naïve airway response to CS. Furthermore, this design allowed us to compare the lung function outcomes between asthmatics and healthy subjects in order to identify the true asthma effect, since there was no alteration to the bronchomotor tone and reflexes caused by long-term treatment with bronchodilators and anti-inflammatory medication.

We found that, immediately after 30 min of CS, levels of eCO increased dramatically (up to 352.7 %) and, as expected, no difference was detected between healthy and asthmatics. According to the calculations provided by the equipment used, the observed changes of eCO corresponded to a significant average increase of carboxyhemoglobin (COHb%), equal in both subgroups. Those findings are compatible with results of other studies investigating the chronic effect of CS on eCO and COHb [22–24] and they confirm that CS is not safe in terms of CO pollution.

In regard to pulmonary function assessment, we avoided pre vs. post CS comparison of spirometric measurements, since it has been shown that the forced maneuvers required may alter the bronchomotor tone [25], influencing IOS [26, 27] and FENO measurements [28]. Furthermore, IOS has been proved more sensitive than spirometry, since changes in IOS-resistance precede changes in PEF and FEV_1_ in experimentally induced airway obstruction [29].

According to our findings, CS caused significant increase of respiratory system total impedance(Z5) in both healthy and asthmatics. This change was attributable to the significant increase of total resistance(R5), since low frequency reactance(X5) values, reflecting respiratory system elastance, were not significantly modified. Furthermore, central airways resistance(R20) increased significantly in both subgroups, which was possibly attributable to the considerable concentrations of thoracic dust (PM_10_) but also inhalable dust (particles diameter ≥1 μm) in cigar smoke [30], normally wiped away by mucociliary clearance in larger airways, before reaching small airways or alveolar space [31, 32]. Frequency dependence of resistance(fdr = R5-R20), which is the most specific indicator of peripheral resistance [33, 34], increased significantly in MA subgroup (+72.1 %), whereas it decreased significantly in HS-subgroup (−50 %), and the difference among the changes was highly significant (*p* < 0.001, Fig. 1a). This indicates that CS affected primarily the peripheral airways in asthmatics, whereas in healthy subjects, dysfunction was located primarily in central airways.

In particular, CS caused significantly higher increase of R20 leading to a significant decrease of fdr in healthy subjects (Table 5, Fig. 1a). The abovementioned pattern has also been reported by Skloot et al. [35], who applied IOS on nonsmokers ironworkers of the Trade Disaster Centre, New York, presenting central airways dysfunction. At the same study [35], smoking population demonstrated the pattern identified in our asthmatics, i.e. higher increase of R5 and increased fdr, which is typical of peripheral airways obstruction, detected in numerous studies applying forced oscillometry on patients with COPD [36–38].

We also found a significant increase of f_res_ and AX and decreased X10 and X20 in asthmatics, but not in healthy smokers. The aforementioned changes are also indicative of acute peripheral airways obstruction [36–38]. In particular, increased fdr, f_res_, and AX, have been proposed as markers of airway closure and express the increased “effective elastance” of the respiratory system [39], but also ventilation inhomogeneity due to peripheral bronchoconstriction [39, 40]. These strongly correlated changes presumably represent the immediate expression of the additive effects of asthma and CS on small airways function.

We also examined the immediate respiratory response to CS in terms of FENO levels for first time. It is known that FENO is immediately reduced after a single cigarette smoking [1, 41], as well as after exposure to sidestream secondhand cigarette smoking [42]. It has been proposed that inhalation of cigarette smoke can acutely reduce FENO through down-regulation of both endothelial and inducible NO synthases [43–45], but also through the rapid conversion of NO to peroxynitrite by reactive oxygen and nitrogen species [44]. We hypothesized that since cigar smoke is rich in nitrogen oxides [10], the above mechanisms could be possibly leading to an acute FENO reduction after CS. However, no significant change of FENO was found, indicating that NO production was not significantly affected by acute inhalation of cigar smoke.

Furthermore, the hypothesis of a different acute response to CS between healthy and asthmatic smokers was also not confirmed in terms of NO production, since there was no difference in FENO changes between MA and HS. In a recent study by Papaioannou et al. [9], the same hypothesis was tested for cigarette smoking. The authors recruited young smokers with and without moderate, well controlled asthma, being treated with long acting β2-agonists and inhaled corticosteroids, and similar results were demonstrated, i.e. no significant FENO alterations in both populations.

Based on the above, it seems that there is no difference regarding the acute respiratory system response to tobacco smoke among healthy and asthmatic smokers, in terms of NO production. Of course, further evaluation of the abovementioned statement is needed through large scale studies.

Our study has several limitations. Firstly, a small number of subjects was examined. Secondly, we didn’t investigate the time duration of the identified effects. Lastly, we didn’t compare the effects of cigar smoking with those of cigarette smoking. However, the purpose of this study was not to suggest that cigar smoking is either safer or more harmful than cigarette smoking. This study aimed to add to the limited evidence supporting that cigar smoking is harmful for human lung and only a single cigar smoking can have significant deleterious effects on the airway function.

## Conclusion

CS has immediate effects on pulmonary function, expressed as ventilation heterogeneity due to peripheral bronchoconstriction in young smokers with mild asthma, and central airways dysfunction in otherwise healthy young smokers. Even though such pathophysiological alterations have no acute clinical expression, they may result in early manifestation of COPD, while clinical status or even FEV_1_ and PEF values may be normal. This hypothesis needs to be tested, especially for asthmatic smokers.

Our results add to the limited evidence that CS is not safe. Given the high prevalence of cigar smoking, especially amongst the youth [11], tobacco prevention policies must become more focused on “alternative” ways of smoking being promoted as more glamourous and safer, and especially on cigar smoking.

## Abbreviations

AX: Reactance area
CO: Carbon monoxide
COHb%: Carboxyhemoglobin %
C: Control group
CS: Cigar smoking
eCO: Exhaled carbon monoxide
fdr: Frequency dependence of resistance
FEF_25%_: Expiratory flow at 25 % of exhaled forced vital capacity
FEF_50%_: Expiratory flow at 50 % of exhaled forced vital capacity
FEF_75%_: Expiratory flow at 75 % of exhaled forced vital capacity
FEF_25–75%_: Expiratory flow at 25 to 75 % of exhaled forced vital capacity
FENO: Fractional exhaled nitric oxide
FEV_1_: Forced expiratory volume at 1 second
f_res_: Resonant frequency
FVC: Forced vital capacity
IOS: Impulse oscillometry system
HS: Healthy smokers
MA: Mild asthma
PEF: Peak expiratory flow
PM_10_: Particulate number 1.0
ppb: Parts per billion
ppm: Parts per million
R5: Respiratory system resistance at 5 Hz
R10: Respiratory system resistance at 10 Hz
R20: Respiratory system resistance at 20 Hz
X5: Respiratory system reactance at 5 Hz
X10: Respiratory system reactance at 10 Hz
X20: Respiratory system reactance at 20 Hz
Z5: Respiratory system total impedance at 5 Hz

## Footnotes

1 Brand of the cigar is not referred intentionally. Manufacturer’s description of the cigar: fermented, non-flavored tobacco, 70 % humidity at 26 °C (80 °F).
